# Integrating fMRI spatial network dynamics and EEG spectral power: insights into resting state connectivity

**DOI:** 10.3389/fnins.2025.1484954

**Published:** 2025-01-28

**Authors:** Souvik Phadikar, Krishna Pusuluri, Armin Iraji, Vince D. Calhoun

**Affiliations:** Tri-Institutional Center for Translational Research in Neuroimaging and Data Science (TReNDS), Georgia State University, Georgia Institute of Technology, and Emory University, Atlanta, GA, United States

**Keywords:** multimodal fusion, simultaneous EEG/fMRI, spatial dynamics, resting state networks, EEG spectra

## Abstract

**Introduction:**

The Integration of functional magnetic resonance imaging (fMRI) and electroencephalography (EEG) has allowed for a novel exploration of the brain’s spatial–temporal resolution. While functional brain networks show variations in both spatial and temporal dimensions, most studies focus on fixed spatial networks that change together over time.

**Methods:**

In this study, for the first time, we link spatially dynamic brain networks with EEG spectral properties recorded simultaneously, which allows us to concurrently capture high spatial and temporal resolutions offered by these complementary imaging modalities. We estimated time-resolved brain networks using sliding window-based spatially constrained independent component analysis (scICA), producing resting brain networks that evolved over time at the voxel level. Next, we assessed their coupling with four time-varying EEG spectral power (delta, theta, alpha, and beta).

**Results:**

Our analysis demonstrated how the networks’ volumes and their voxel-level activities vary over time and revealed significant correlations with time-varying EEG spectral power. For instance, we found a strong association between increasing volume of the primary visual network and alpha band power, consistent with our hypothesis for eyes open resting state scan. Similarly, the alpha, theta, and delta power of the Pz electrode were localized to voxel-level activities of primary visual, cerebellum, and temporal networks, respectively. We also identified a strong correlation between the primary motor network and alpha (mu rhythm) and beta activity. This is consistent with motor tasks during rest, though this remains to be tested directly.

**Discussion:**

These association between space and frequency observed during rest offer insights into the brain’s spatial–temporal characteristics and enhance our understanding of both spatially varying fMRI networks and EEG band power.

## 1 Introduction

**Figure 1:**
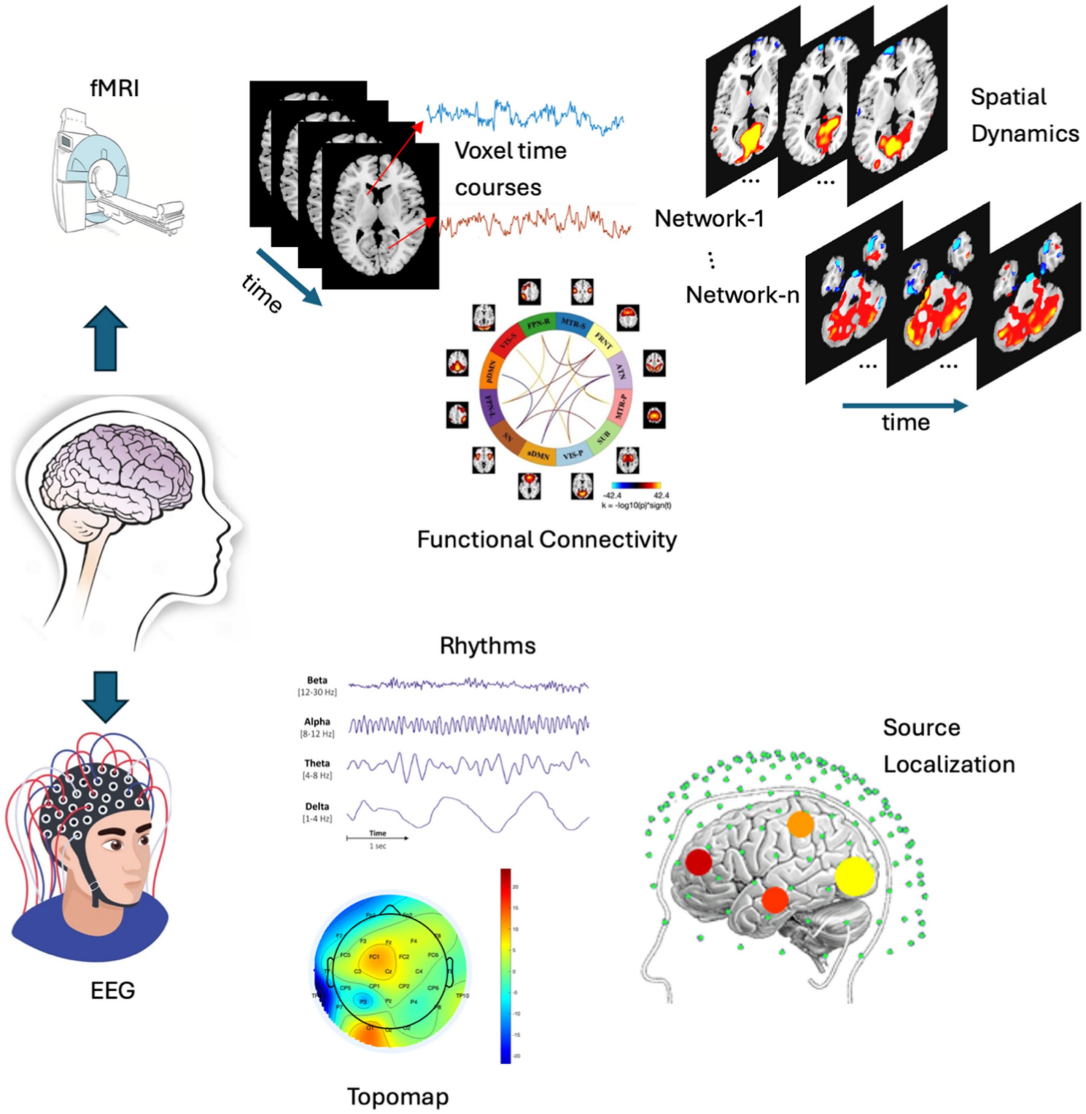
A comparative example is presented, showcasing the analysis of fMRI (top) and EEG (bottom). The fMRI analysis includes voxel time courses, spatial dynamics and functional connectivity, while the EEG analysis features topographic maps, various EEG rhythms, and source localization. These are typical analyses (though not limited to these) performed on both modalities, which are then integrated to conduct a joint analysis. (N.B. Few elements of this figure such as EEG, Source localization, etc were collected from google image search).

Integrating two or more brain imaging techniques is rapidly advancing the analysis of functional connectivity, revealing a deeper understanding of the brain at a large scale (Allen et al., 2018; Bridwell and Calhoun, 2019; Calhoun et al., 2007). Different brain imaging techniques capture unique features of brain function and complement each other’s limitations, providing a comprehensive view of the brain. For instance, functional magnetic resonance imaging (fMRI) indirectly measures spontaneous neural activity via the blood oxygenation level-dependent (BOLD) signal, offering good spatial resolution (1–3 mm) but relatively poor temporal resolution (1–3 s) (Glover, 2011). Another widely used approach, electroencephalography (EEG), records the electrical activity of groups of neurons, providing high temporal resolution (1–10 ms), yet its spatial resolution limits precise anatomical understanding of underlying neural sources (Yu et al., 2016). Therefore, fMRI and EEG offer complementary imaging signals, and merging data collected simultaneously offers a particularly beneficial method for studying brain dynamics across a wide range of spatial and temporal scales (Chang and Chen, 2021; Jorge et al., 2014; Mulert, 2013; Philiastides et al., 2021). However, the challenges remain on how to link the electrical (EEG) and hemodynamic response (fMRI) to each other during different states of brain dynamics. EEG has high temporal resolution and low spatial resolution, whereas fMRI has high spatial resolution and low temporal resolution (a comparative example is shown in Figure 1). Challenges remain in fusing these two modalities in real time.

Multimodal data fusion, a powerful approach for combining different modalities of brain imaging (as well as other body imaging modalities), refers to a broad range of data-driven approaches to explore the insights gained from two or more modalities. It is widely used in simultaneous EEG/fMRI studies, and can be implemented via a variety of approaches, e.g., independent component analysis (ICA), linear regression, and hybrid methods (Akhonda et al., 2018; Allen et al., 2018; Bridwell and Calhoun, 2019; Calhoun and Sui, 2016; Heugel et al., 2022; Mangalathu-Arumana et al., 2018; Mosayebi and Hossein-Zadeh, 2020; Stephen et al., 2014; Wirsich et al., 2020). Such approaches allow us to merge EEG and fMRI into a common feature space. For example, we can generate spatial–temporal independent components, which can serve as biomarkers for distinguishing schizophrenia from healthy controls (Calhoun et al., 2007). Alternatively, some techniques (Muraskin et al., 2018) use encoding models to link EEG and fMRI by learning the optimal mapping between feature representations of the two modalities.

In many fMRI studies, a common assumption is that brain networks remain fixed in their spatial configuration during a typical scan. This assumption overlooks the highly spatial dynamic nature of brain networks which can undergo spatial changes via expansion or shrinking over time, in addition to the dynamical changes in functional connectivity (Iraji et al., 2024; Phadikar et al., 2024; Pusuluri et al., 2024). A recent study explored how these spatial dynamic subspaces capture unique disruption in brain networks associated with schizophrenia, which vary by sex (Iraji et al., 2024). These disruptions involve transient overlaps in networks and are potentially linked to genetic risk factors for the disorder. However, the temporal resolution of these spatially varying brain networks was not studied well. Here for the first time, we analyzed the temporal resolution of these spatially varying brain networks by integrating fMRI with EEG. Spectral power of EEG is widely used to understand electrical activity produced by the brain and can provide insights into various brain states and functions. The frequency bands of EEG are generally grouped as delta (0.5–4 Hz), theta (4–8 Hz), alpha (8–13 Hz), beta (13–30 Hz), and gamma (30–100 Hz) waves. Each band is associated with different states of brain function and cognitive processes (Talebi et al., 2022; Zhang et al., 2023). In many EEG studies, band powers are analyzed over a range of fixed time-period and ignored the time-resolved band power. They also undergo temporal changes over time. In this study, we consider time-varying EEG band power and link them with spatially varying resting state fMRI networks.

While prior studies (Allen et al., 2018; Bridwell and Calhoun, 2019; Chang et al., 2013) have found EEG coupling to spatially fixed brain networks. Here we study, for the first time, the relationship of voxel wise and volume wise changes of different brain networks in resting fMRI data to time-varying band power in concurrently collected EEG data. We computed the spatial dynamics of the fMRI brain networks using sliding window-based spatially constrained ICA (scICA) (Iraji et al., 2021, 2024; Phadikar et al., 2024) and then evaluated the coupling between these spatial dynamic networks with time varying EEG band power during the resting state. To reduce the number of statistical comparisons, we first characterized the spatial dynamics of fMRI networks by measuring the volume of each network (whether shrinking or expanding) and how it is related to the various frequency bands of EEG during the brain at rest. Further, we analyzed these networks at the voxel-level and evaluated the coupling of each voxel with time-varying EEG band power. However, to our knowledge, no work has yet combined time-varying EEG band power with fMRI spatial dynamic networks. The objective of this study is to (1) develop a multimodal data fusion technique to link EEG with fMRI, (2) volumetric analysis of spatial dynamic fMRI networks, and (3) Analyzing temporal and spatial resolution of resting state brain networks. Our work thus focuses on the relationship of the spatial dynamics of brain networks in fMRI to time variation in four well studied EEG frequency bands. The space-frequency connectivity observed in the resting state may reveal information about how brain networks interact and process information during rest.

## 2 Methods

**Figure 2:**
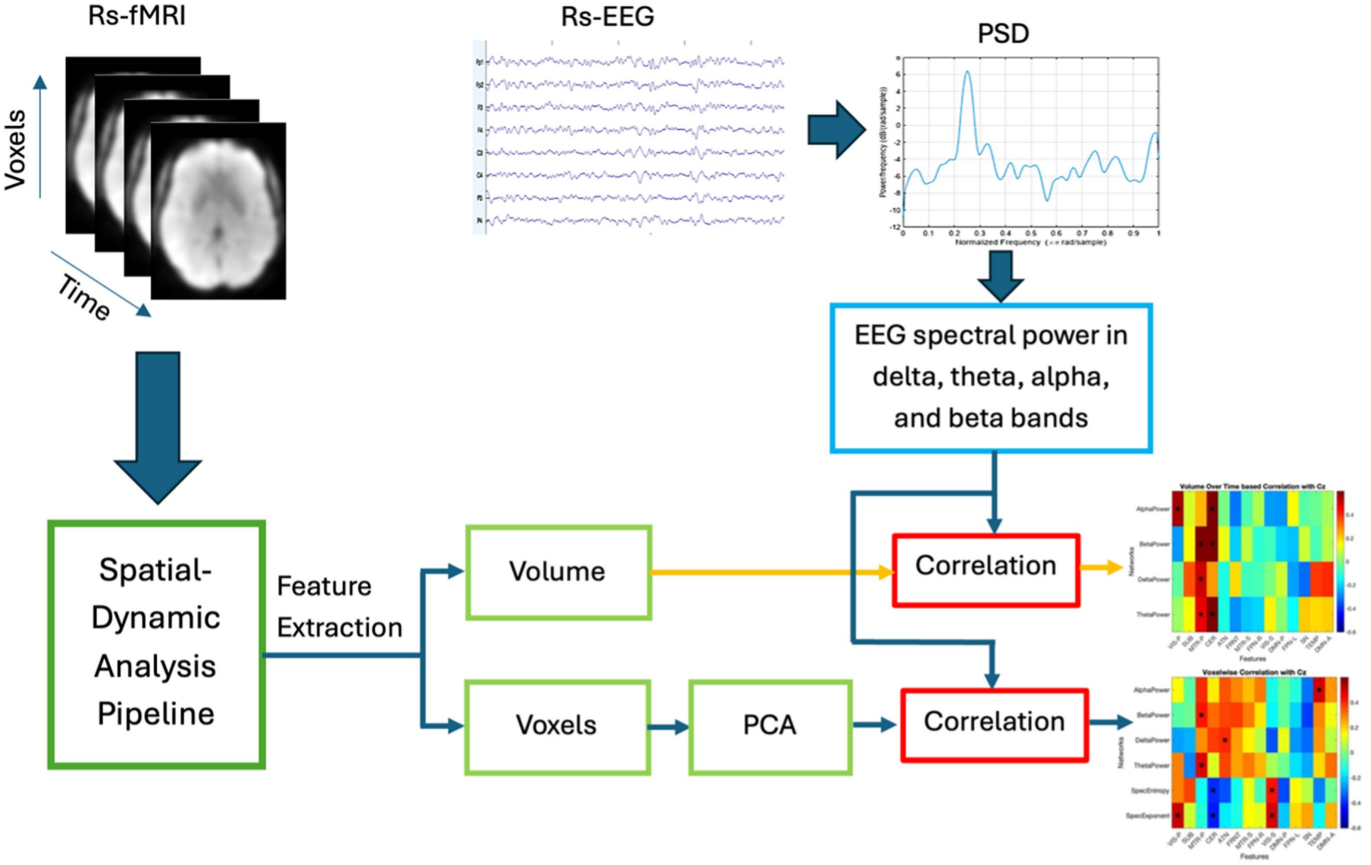
Basic pipeline of the proposed multi-modal fusion model. The volume of rs-fMRI networks is computed over sliding windows as the number of voxel activity above a statistically evaluated threshold (the threshold for individual networks were obtained through a separate statistical analysis—explained below). On the other hand, four band powers are computed from the EEG over a sliding window. Finally, volume-based correlation and voxel-wise correlation with four time-varying EEG band powers are computed and the correlation maps are interpreted.

This section describes the proposed multi-modal approach for the fusion of EEG and fMRI with the model pipeline presented in Figure 2. The proposed technique is described in following steps: (1) *spatial dynamics of rs-fMRI*—we estimated spatial maps (SMs) using a sliding window based scICA from rs-fMRI data; (2) *power spectrum of EEG*—time varying four band powers (delta, theta, alpha, and beta) were obtained using sliding window approach; (3) *fusion analysis and Interpretation*—we computed the volume of each spatial map as the number of voxels with activity level greater than statistically evaluated threshold (V_TH_ = 0.5, 1.0, 1.5,…, 3.5), and subsequently we measured the correlation between time-varying EEG band power and time-varying volume of the fMRI network as well as time-varying voxel activity. Further, we evaluated the correlation at voxel level. Detailed explanations are presented in the following subsections.

### 2.1 Spatial dynamics of rs-fMRI

**Figure 3:**
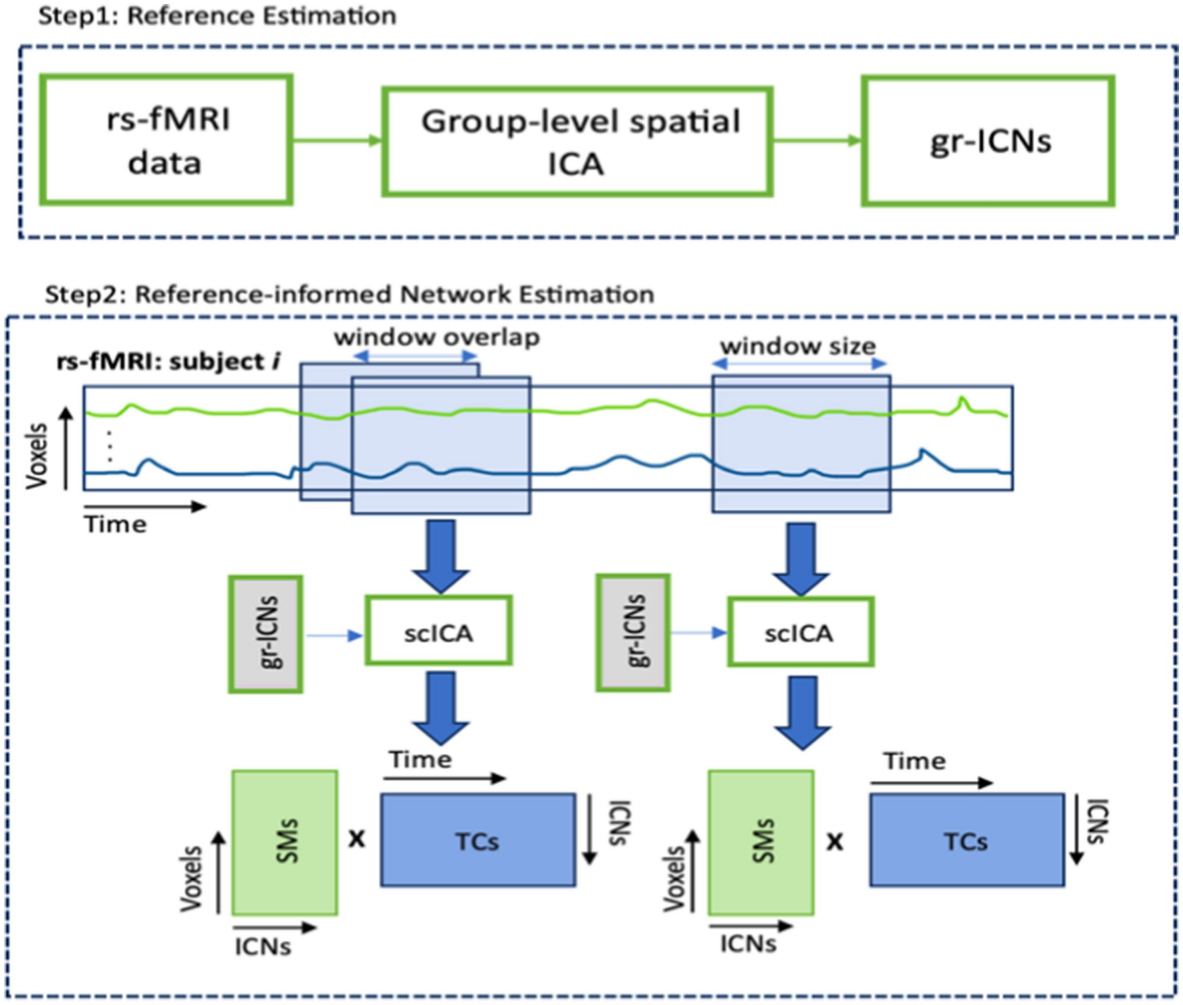
Basic pipeline of computing spatial maps (SMs) and time courses (TCs) from rs-fMRI data. Step 1 applies group-level spatial ICA on resting state fMRI data and produces group-level intrinsic connectivity networks (gr-ICNs). Step 2 applies the MOO-ICAR over sliding windows to rsfMRI of each subject using previously generated gr-ICNs as a reference.

The spatial dynamic analysis was performed on rs-fMRI data, using the following steps (see Figure 3). We utilized the GIFT toolbox^1^ (Calhoun et al., 2001; Iraji et al., 2021) to extract large-scale brain networks (or group ICNs) from the resting-state functional images, following the procedure described in Iraji et al. (2019). Next, we employed a sliding-window approach to each subject and applied a spatially constrained ICA approach called multivariate-objective optimization ICA with reference (MOO-ICAR) (Du and Fan, 2013) to estimate the time-resolved networks corresponding to the previously identified gr-ICNs. We used a model order of 20 to identify large-scale networks in the MOO-ICAR model, as suggested by Iraji et al. (2016, 2024). MOO-ICAR has proven to be highly effective in large-scale brain network estimation for varying data lengths and is noise-resistant (Iraji et al., 2023). A tapered window made by convolving a rectangle (width = 30 × TR seconds) with a Gaussian (s = 6 s) and sliding step size of 2 s was used to implement the commonly used sliding-window technique (Allen et al., 2014). Subject-specific spatial maps (SMs) and timecourses (TCs) were obtained for each window. These resulting spatial maps carry the spatial information (e.g., Spatial distribution) of any functional network.

### 2.2 Power spectrum of EEG

A sliding window of the same size (30 × TR seconds) as in rs-fMRI analysis was applied to the EEG data, and Welch’s method was utilized to estimate the power spectral density (PSD). Subsequently, band power ( $BP_{\mathrm{elect}}^{M}\left(w\right)$ ) from three midline electrodes (elect = Cz, Fz, Pz) in four EEG bands (M = delta, theta, alpha, beta) were derived from the PSD for each time-window ( w ). Band powers were computed from three midline electrodes (Fz, Cz, PZ), centrally placed over the head from inion to nasion using the 10–20 electrode placement system. This setup captures a broad power spectrum from both the left and right hemispheres (Calhoun et al., 2007). We selected band power over other EEG measures because different EEG frequency bands are associated with various brain states and function (Talebi et al., 2022), play a key role in diagnosing various neurological and psychiatric conditions (Bradley et al., 2024), and are essential for understanding the neural underpinnings of various cognitive process.

### 2.3 Fusion analysis

Our goal is to link EEG and fMRI and investigate whether variations in neural activity patterns (from EEG) synchronize with the spatially varying resting-state fMRI networks. We conducted the following experiments to evaluate this, as described below.

#### 2.3.1 Voxel-wise fusion

The size of the voxels in each spatial map is significantly larger compared to the size of the band power. For example, in our experiment, the number of voxels in the primary visual network is (68,235 × w), whereas the number of alpha band power values is 1 × w. Correlating these across the time-window (w) could lead to inaccurate information due to this unequal dimensionality. To address this, we first applied principal component analysis (PCA) on the (voxels × w) data of each network and obtained the first principal component and subsequently, computed the correlation (X) with time-varying band powers ( $BP_{\mathrm{elect}}^{M}\left(w\right)$ ) across the time-window (w), as described in Equation 1.


$$X_{\mathrm{Nnets},\mathrm{elect}}^{M}=\mathrm{correlation}\;\left(PCA\left(SM_{\mathrm{Nnets}}\left(t\right)\right),\;BP_{\mathrm{elect}}^{M}\left(t\right)\right)$$ (1)

Where, Nnets=1,2,…,14 refers to the number of resting state fMRI networks (it is 14 in our study, described in result section), electrode and M indexes three midline electrodes (Cz, Fz, Pz) and the four EEG bands (delta, theta, alpha, and beta) respectively.

#### 2.3.2 Volume-based fusion

We computed the volume of the network and its variation across time-window ( $Vol_{\mathrm{Nnets}}\left(w\right)$ ) and correlated with the spectral power in four EEG bands across w. The volume was calculated by counting the number of voxels above statistically evaluated threshold $V_{TH}=\left(Z=0.5,1.0,1.5,\ldots ,3.5\right)$ as described in Equations 2, 3. The statistical *t*-test was performed to select appropriate threshold for each network is described in result section.


$$Vol_{\mathrm{Nnets}}\left(t\right)=\overset{N}{\underset{i=1}{\Sigma }}\delta \left(v_{i}\left(w\right)>V_{TH}\right)$$ (2)

$$Y_{\mathrm{Nnets},\mathrm{elec}}^{M}=\mathrm{correlation}\;\left(Vol_{\mathrm{Nnets}}\left(w\right),\;BP_{\mathrm{elec}}^{M}\left(w\right)\right)$$ (3)

Where, $Vol_{\mathrm{Nnets}}\left(w\right)$ is the volume of active voxels in $SM_{\mathrm{Nnets}}\left(w\right)$ at time-window t , N is the total number of voxels in $SM_{\mathrm{Nnets}}\left(w\right)$ , $v_{i}\left(w\right)$ represents the activity level of voxel i at time-window w . $\delta \left(\text{.}\right)$ is the indicator function, which equals 1 if the condition inside is true, and 0 otherwise.

The subject-specific correlation was calculated for both the fusion. For an example, there are 14 networks and four bands, resulting the correlation matrix (X) of size (14 × 4) per electrode. If we consider Z number of subjects in our dataset, and compute the correlation analysis for each of them, the resulting correlation matrix would have size of (14 × 4 × Z). PCA was then applied across Z to further reduce the data. The first principal component of dimension (14 × 4) was obtained and interpreted for our analysis.

### 2.4 Dataset description

The proposed multi-modal approach was performed on simultaneously collected EEG and fMRI data of 90 subjects selected from two publicly available datasets (Gu et al., 2023; Telesford et al., 2023), a third dataset previously collected at the Mind Research Network (Wu et al., 2010), and a fourth dataset collected from an ongoing project at the Center for Advanced Brain Imaging (CABI). The details of datasets are presented in Table 1. For more details of these publicly available datasets can be found in their respective manuscripts (Gu et al., 2023; Telesford et al., 2023; Wu et al., 2010), to which readers may refer. Here, we present the specifics of our ongoing experimental dataset. The ongoing experiment comprised simultaneous EEG-fMRI recording sessions, during which participants were instructed to remain still, awake, and relaxed inside a dimly lit scanner room. Each scanning session included a 7-min structural MRI scan followed by two 7-min rs-fMRI scans. Three task functional MRI scans lasting 6 min each were conducted while subjects performed a reading task. Functional images were acquired using a whole-body 3 T Siemens PRISMA Fit MR system at the CABI. Simultaneous EEG was recorded using a 32-channel BrainAmp MR-compatible system. A Brain Products R-Net MR cap was applied to the scalp with electrodes positioned according to the international 10–20 system, and an electrocardiogram (ECG) was recorded from an electrode applied to the participant’s back. The EEG was synchronized to the scanner clock using a Brain Products SyncBox, and scanner triggers were recorded with the use of a Brain Products TriggerBox. The analysis for the current study was conducted on resting state data.

**Table 1:** Simultaneous EEG/fMRI dataset description.

Parameters		Dataset (Telesford et al., 2023)	Dataset (Gu et al., 2023)	Dataset (Wu et al., 2010)	Inhouse dataset
Number of participants		22	33	25	10
Resting state duration (s)		600	600	420	420
fMRI	TR (ms)	2,100	2,100	2000	2000
	TE (ms)	24.6	25	39	30
	Matrix size	64 × 64	NA	64 × 64	64 × 64
	Voxel size (mm)	3.4 × 3.4 × 3.3	3 × 3 × 3	3.5 × 3.5 × 3.5	3.4 × 3.4 × 4
	Slices	38	35	27	32
EEG	Number of channels	64	32	32	32
	Impedances (kOhm)	< 20	<20	<5	<5
	Sampling rate (KHz)	5	5	5	5

### 2.5 Pre-processing simultaneous EEG/fMRI data

The resting state continuous EEG data was pre-processed via EEGLAB^2^ toolbox in MATLAB.^3^ The scanner artifacts were removed using FASTR algorithm within FMRIB toolbox.^4^ Next, the EEG data were down sampled to 1 kHz and bandpass filtered within the range of 1 to 30 Hz. The cardiac artifacts were identified and removed with the FMRIB toolbox, and the data were re-referenced to the common average. Next, the EEG data was decomposed into independent components (ICs) using temporal ICA to identify and eliminate residual pulse, muscle, and eye movement artifacts. Three midline electrodes (Fz, Cz, Pz) were selected for further investigation because served as the best channels to detect both left and right sources. However, we had to exclude the channel “Oz” as it is not available in Brain products MR compatible R-Net cap. The resting state fMRI data were pre-processed in SPM12,^5^ and the preprocessing steps encompass despiking, realignment, spatial normalization into MNI space, and blurring with adaptive kernel to a desired smoothness with a full width at half maximum (FWHM) of 6 mm as described in Allen et al. (2018).

## 3 Results

### 3.1 Spatial dynamics fMRI networks

**Figure 4:**
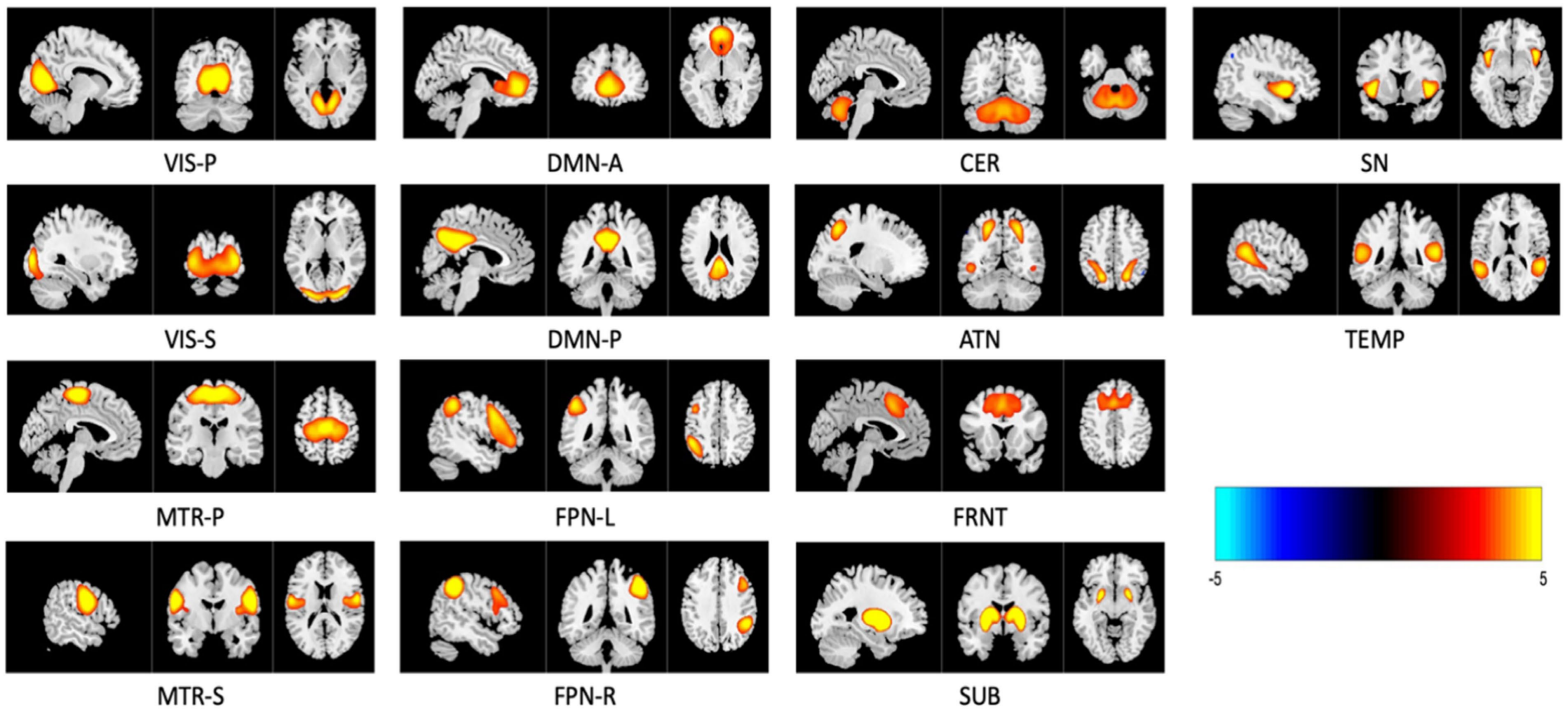
Three planar cross-section (sagittal, coronal, and transverse) image representations of 14 relevant intrinsic connectivity networks (ICNs) sliced at their highest voxel activity. These maps are z-scored and only display regions with z≥2.5 , with anatomical images overlaid in the background. Networks are named as VIS-P/S (visual primary/secondary), MTR–P/S (Somatomotor primary/secondary), DMN-A/P (default mode anterior and posterior), FPN-L/R (frontoparietal left/right), SUB (subcortical), CER (cerebellar), ATN (attention—dorsal), FRNT (frontal), SN (salience), and TEMP (temporal).

Twenty components were generated after applying group-level spatial ICA on rs-fMRI data. Out of the 20, 14 were identified as intrinsic connectivity networks (ICNs), while others were excluded as they were deemed noise-related brain networks based on their spatial–temporal characteristics and insights from previous studies (Iraji et al., 2019). The activity maps of these 14 relevant brain networks are shown in Figure 4. These networks are labeled as VIS-P/S (visual primary/secondary), MTR-P/S (Somatomotor primary/secondary), DMN-A/P (default mode anterior and posterior), FPN-L/R (frontoparietal left/right), SUB (subcortical), CER (cerebellar), ATN (attention-dorsal), FRNT (frontal), SN (salience), TEMP (temporal). Next, time-resolved subject-specific SMs and TCs were computed using MOO-ICAR applied through a sliding-window approach corresponding to the previously identified 14 ICNs (gr-ICNs) (Iraji et al., 2024). The subject-specific SMs (voxels per network per time-window) were z-scored and used for the further analysis.

### 3.2 Selection of thresholds (V_TH_) for volume of fMRI networks

**Figure 5:**
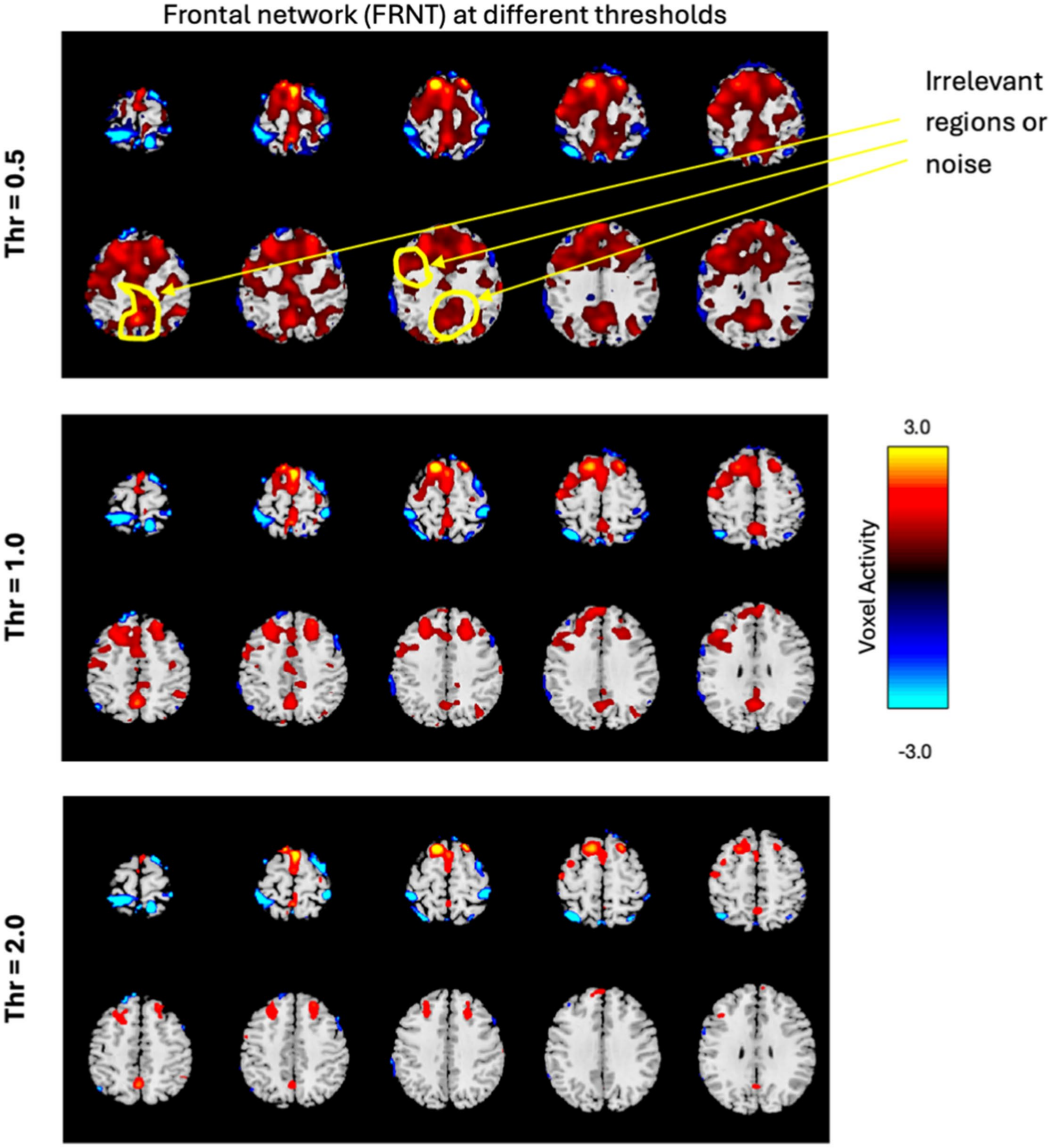
The frontal network is displayed in few slices, at three different thresholds, with the color bar representing activity levels ranging from −3.0 to +3.0. The figure illustrates how varying thresholds impact the regions of the network, highlighting the importance of selecting an appropriate threshold.

The volume of a network measures the relevant regions of the network and is determined by the total number of voxels with activity greater than or equal to the threshold (V_TH_), as described in Equation 2. To precisely capture the relevant regions of the network and avoid including other activities, we computed the volume using a set of thresholds ranging from 0.5 to 3.5 with a step size of 0.5 (since the SMs were converted into z-scores). A lower threshold may include noise, while a higher threshold could reduce the relevant regions of the networks. For example, Figure 5 shows the frontal network at three different thresholds. If we compare the original frontal network shown in Figure 4, it’s clear that, the lower threshold includes irrelevant regions (noise), and as the threshold increases, the network volume shrinks. Therefore, selecting an appropriate threshold is crucial. Additionally, the same threshold may not be optimal for all brain networks.

This is in line with the analysis performed in Pusuluri et al. (2024) which found that multiple thresholds can be employed to study complex dynamics of fMRI networks including their altered spatial focus of activity, altered spatial dynamism, and how altered focus of activity is associated with various cognitive, symptom, and drug scores of subjects. Pusuluri et al. (2024) found that networks showed disparate dynamics and significance at varying thresholds and therefore, we performed our analysis across all these thresholds as well.

The goal here is to select a standardized and automated thresholding approach that is also subject specific (similar to a Z-score). We then interpret the group level results, including evaluating their biological plausibility. To determine the appropriate threshold, we followed these steps: (1) Computed spatial maps using the method described in Figure 3; (2) Calculated the volume for each threshold (V_TH_ = 0.5, 1.0, 1.5, …, 3.5); (3) Averaged the four band powers (delta, theta, alpha, and beta) for each electrode; (4) Computed the correlation between the volumes calculated for each threshold and the average band power of each electrode; (5) Generated a correlation matrix for all subjects in our dataset; (6) Performed a one sample t-test on the correlation matrix across subjects; (7) Conducted a multiple comparison test to select the appropriate threshold using a 5% significance level. The one-sample t-test was conducted on the correlation matrix for three electrodes separately to observe performance variance across electrodes. The *p*-values for each threshold are presented in Tables 2–4. The threshold was selected at *p* < 0.05 from the multiple comparison test. From Tables 2–4, it can be observed that the p-values of VIS-P/S, SUB, MTR-P/S, CER, ATN, FPN-R, and DMN-A networks are less than 0.05 at V_TH_ = 2.5, 2.0, 3.0, 2.5, 2.0, 2.0, 1.5, respectively, (indicated in bold red). Additionally, the *p*-values are mostly consistent across the three electrodes. However, other brain networks do not show a significant correlation between network volume and average band power, as their *p*-values are greater than 0.05. We selected specific thresholds for the respective networks and continued with further analysis.

**Table 2:** One sample *t*-test results to determine if the mean correlation coefficient is significantly different from zero across all the subjects.

	V_TH_ = 0.5	V_TH_ = 1.0	V_TH_ = 1.5	V_TH_ = 2.0	V_TH_ = 2.5	V_TH_ = 3.0	V_TH_ = 3.5
VIS-P	0.2208	0.2141	0.1668	0.0583	**0.0280**	0.0355	0.1156
SUB	0.0469	0.0569	0.1199	0.6299	0.0575	**0.0329**	0.0442
MTR-P	0.0887	**0.0415**	0.2315	0.5806	0.1091	0.0714	0.0763
CER	0.0021	0.0028	0.0074	0.6421	**0.0015**	0.0392	0.0812
ATN	0.0670	0.0572	0.6262	**0.0048**	0.0633	0.1049	0.1288
FRNT	0.4280	0.2870	0.9353	0.4010	0.4037	0.5809	0.6663
MTR-S	0.3395	0.2597	0.1712	0.8221	0.2567	0.1531	0.1250
FPN-R	0.3690	0.5121	0.4079	0.3805	0.3545	0.5616	0.6115
VIS-S	0.2465	0.1765	0.1448	0.0450	0.3744	0.1368	0.0720
DMN-P	0.2238	0.2425	0.1702	0.4166	0.1856	0.1587	0.1640
FPN-L	0.6328	0.7625	0.8635	0.7486	0.7346	0.7475	0.7271
SN	0.1743	0.1268	0.0760	0.6218	0.0825	0.0869	0.1291
TEMP	0.6491	0.6231	0.0961	0.2348	0.6157	0.4816	0.4456
DMN-A	0.0569	0.0651	**0.0009**	0.0094	0.0741	0.0060	0.0028

*p*-values are shown here for all 14 networks and 7 thresholds for Cz electrode. The V_TH_ for the respective network is selected which scored the lowest of *p* < 0.05 (bold in red). For example, V_TH_ = 1.5, and V_TH_ = 2.5 showed the highest significance for DMN-A and CER, respectively.

**Table 3:** One sample *t*-test results to determine if the mean correlation coefficient is significantly different from zero across all the subjects.

	V_TH_ = 0.5	V_TH_ = 1.0	V_TH_ = 1.5	V_TH_ = 2.0	V_TH_ = 2.5	V_TH_ = 3.0	V_TH_ = 3.5
VIS-P	0.1969	0.1984	0.1306	0.6323	0.4129	0.0925	0.0843
SUB	0.0099	0.0057	0.1116	0.3251	0.0043	**0.0043**	0.0056
MTR-P	0.0326	**0.0141**	0.1061	0.9848	0.0433	0.0262	0.0275
CER	0.0109	0.0238	0.0027	0.4215	**0.0016**	0.0020	0.0038
ATN	0.0164	0.0179	0.0504	**0.0034**	0.0143	0.0203	0.0237
FRNT	0.6894	0.4508	0.6485	0.5626	0.5424	0.7708	0.8865
MTR-S	0.0825	0.0597	0.0419	0.9426	0.0975	0.0233	**0.0115**
FPN-R	0.7279	0.8751	0.6312	0.4486	0.7555	0.9544	0.9974
VIS-S	0.1355	0.1006	0.0688	**0.0183**	0.2887	0.0623	0.0277
DMN-P	0.4993	0.5161	0.4376	0.4242	0.4941	0.4262	0.4171
FPN-L	0.3935	0.5275	0.4969	0.8496	0.4692	0.5617	0.5968
SN	0.4287	0.3080	0.2455	0.6286	0.2343	0.2111	0.2483
TEMP	0.9487	0.9463	0.4289	0.0758	0.9273	0.6893	0.6145
DMN-A	0.0003	0.0005	**0.0001**	0.0076	0.0010	0.0020	0.0010

*p*-values are shown here for all 14 networks and 7 thresholds for Fz electrode. The V_TH_ for the respective network is selected which scored the lowest of *p* < 0.05 (bold in red). For example, V_TH_ = 3.0, and V_TH_ = 1.0 showed significance for SUB and MTR-P, respectively.

**Table 4:** One sample *t*-test results to determine if the mean correlation coefficient is significantly different from zero across all the subjects.

	V_TH_ = 0.5	V_TH_ = 1.0	V_TH_ = 1.5	V_TH_ = 2.0	V_TH_ = 2.5	V_TH_ = 3.0	V_TH_ = 3.5
VIS-P	0.0596	0.0545	0.0741	0.1753	0.2628	0.5763	0.5518
SUB	0.2490	0.2397	0.2692	0.4890	0.1824	0.1661	0.1667
MTR-P	0.1931	0.1175	0.3130	0.8705	0.1813	0.1031	0.1097
CER	0.0049	**0.0039**	0.0158	0.9805	0.0130	0.0103	0.0099
ATN	0.0046	0.0055	0.3087	**0.0011**	0.0051	0.0093	0.0122
FRNT	0.8583	0.5921	0.4864	0.8654	0.8580	0.9987	0.9313
MTR-S	0.3532	0.2413	0.1375	0.5447	0.2818	0.0813	0.0470
FPN-R	0.5122	0.5823	0.8379	**0.0391**	0.5701	0.8595	0.9087
VIS-S	0.4207	0.2772	0.2508	0.1673	0.2424	0.2558	0.1666
DMN-P	0.2852	0.2810	0.3672	0.8804	0.2759	0.3108	0.3404
FPN-L	0.5924	0.6919	0.9550	0.3765	0.5501	0.9092	0.9980
SN	0.9654	0.7877	0.8713	0.4280	0.6812	0.7764	0.8351
TEMP	0.8265	0.8520	0.2687	0.1340	0.9449	0.8618	0.7873
DMN-A	0.0410	0.0411	**0.0013**	0.0068	0.0489	0.0034	0.0013

*p*-values are shown here for all 14 networks and 7 thresholds for Pz electrode. The V_TH_ for the respective network is selected which scored the lowest of *p* < 0.05 (bold in red). For example, V_TH_ = 1.0, and V_TH_ = 2.0 showed significance for CER and ATN, respectively.

### 3.3 Spatially varying brain networks vs. temporally varying EEG band power

**Figure 6:**
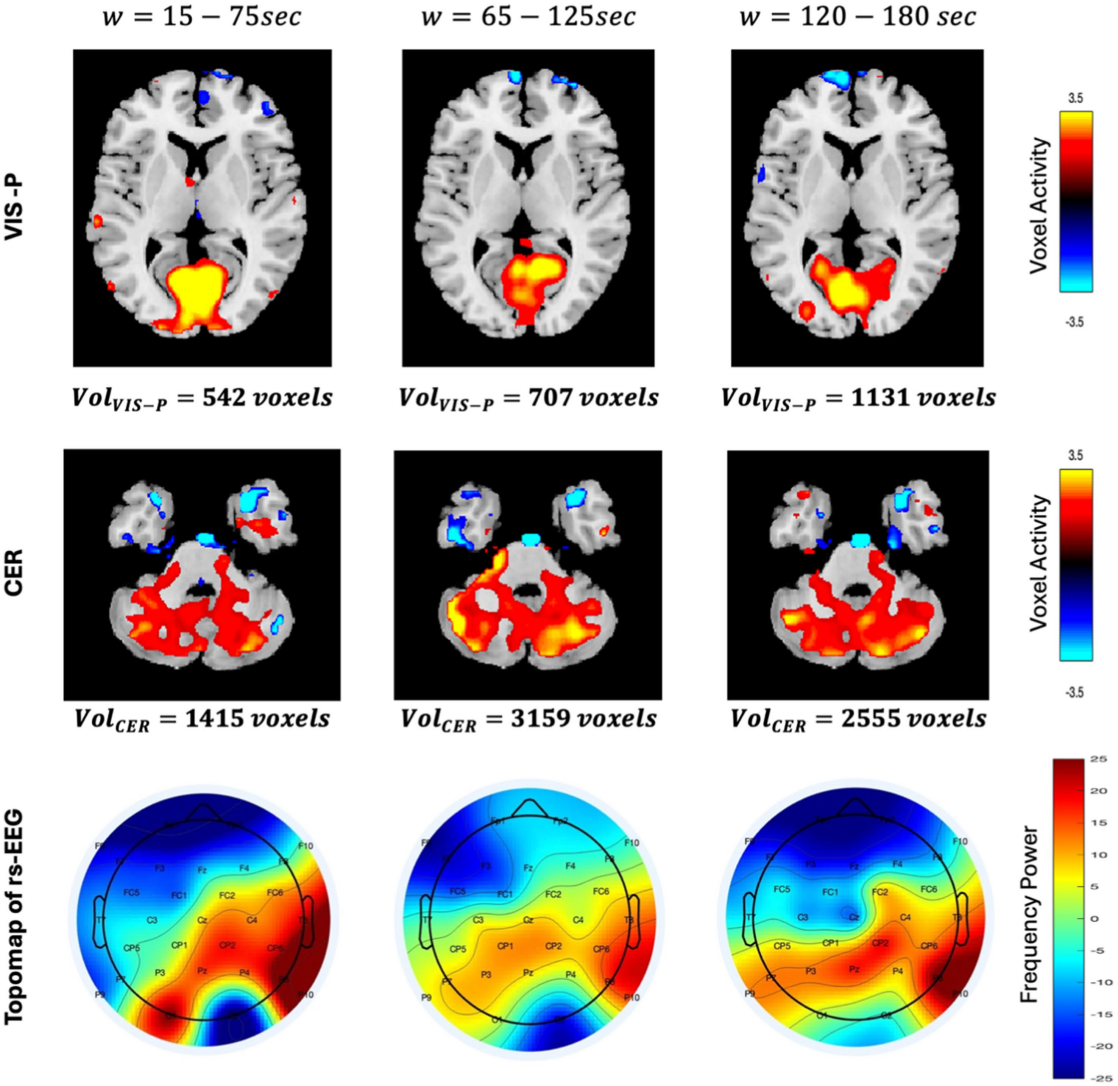
Spatial differences in VIS-P and CER networks are shown here in axial view for three different time windows w=15−75,65−125,120−180 . Activity maps are displayed with z≥2.5 using GIFT toolbox (http://trendscenter.org/software/gift). Color bar for spatial maps represent the activity level ranging from −3.5 to +3.5. Similarly, the topo maps (describing the spatial distribution of electrical activity on the scalp) of rs-EEG are displayed for three time-windows. Color bar of the topo map represents the power of the frequency ranging from −25 to +25.

We measured the spatial dynamic characteristics by computing the volume (using V_TH_ from Tables 2–4) at each window to examine if the network is shrinking or expanding. For example, the activity maps of the primary visual and cerebellar networks at three different time windows are shown in Figure 6. It is evident from the figure that the volume of the primary visual network is 542, 707, and 1,131 voxels at time-window w = 15–75 s, 65–125 s, and 120–180 s, respectively. This may indicate that the primary visual network expanded over time during rest. Similarly, the cerebellar network initially expanded in volume (from 1,415 voxels to 3,159 voxels) and then shrunk over time (from 3,159 voxels to 2,555 voxels). Activity maps are displayed with Z ≥ 2.5 for all three time-windows for both the networks.

Additionally, it is observed that the voxel-level activity is not uniform across the entire network. In the primary visual network, voxel activities are initially higher (the color bar on the right shows the magnitude of the voxel’s activity, with cyan indicating negative activity, yellow indicating the highest positive activity, and at the middle, black representing no activity) throughout the entire network region. However, as the volume increases (at w = 65–125 s), this highest positive activity (yellow) shrinks to the top of the network and eventually shifts to the left side. This observation clearly indicates that voxel activity is not uniform across the network’s region, instead they vary over time. This phenomenon motivated us to link this time-varying voxel activity with time-varying EEG band power. The topographical plot at three different time-windows is shown in Figure 6 (third row) to provide an approximate (large-scale) spatial distribution of neuronal activity using channel locations. We assume the midline electrode Pz is close to the VIS-P and CER networks, and it is evident from the figure that the neural activity level decreases initially and then increase over time around Pz (observed by the color contrast of the topographical map representing the power of the frequency). The observed spatial variation in fMRI networks and temporal variation in EEG band power encourage us to investigate association between them, which may help in gaining a deeper understanding of brain dynamics.

### 3.4 Volume based fusion

**Figure 7:**
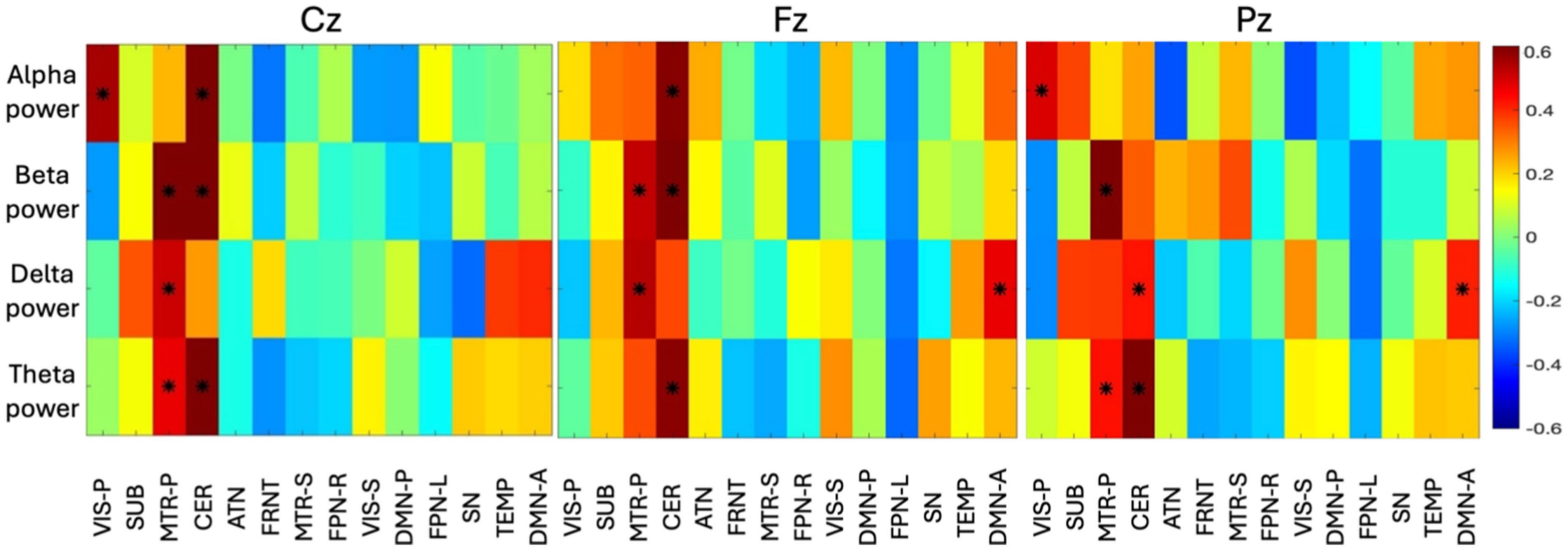
The group-level correlation map between four time-varying band power and time-varying volume of 14 networks from all the subjects are displayed here (correlation > abs|0.4| are identified with an asterisk). The principal component of all the subject-specific correlation maps is shown as group-level correlation map. Three different correlation maps are presented for three different electrodes (Cz, Fz, Pz). The color bar indicates the correlation value ranging from −0.6 to +0.6.

In the volume-based fusion analysis, the resultant correlation matrix shows a pattern where networks are found to be correlated with band power and presented in Figure 6. Three correlation maps were computed separately for all three midline electrodes (Cz—left, Fz—center, Pz—further right), describing a sweet spot where space and frequency are found to be synchronized. A correlation > absolute |0.4| was selected as a significant correlation and identified with an asterisk in the correlation map shown in Figure 7. We selected a correlation threshold of abs |0.4| as significant because, with our sample size of 90, it corresponds to an uncorrected *p*-value of 0.0001 which passes Bonferroni correction for multiple comparisons. In Figure 7, electrode Pz reveals distinct frequency localizations across networks: alpha power strongly correlates with the primary visual network (VIS-P), beta power with the primary somatomotor network (MTR-P), delta power with both CER and DMN-A, and theta power with MTR-P and CER. Conversely, Fz and Cz exhibit limited localizations of the four EEG bands across networks. At both Fz and Cz, alpha, beta, and theta show high correlations with CER, while beta and delta correlate with MTR-P. Additionally, at Cz, alpha is highly linked to the VIS-P network, while delta is prominent in the DMN-A network at Fz.

### 3.5 Voxel-wise fusion

**Figure 8:**
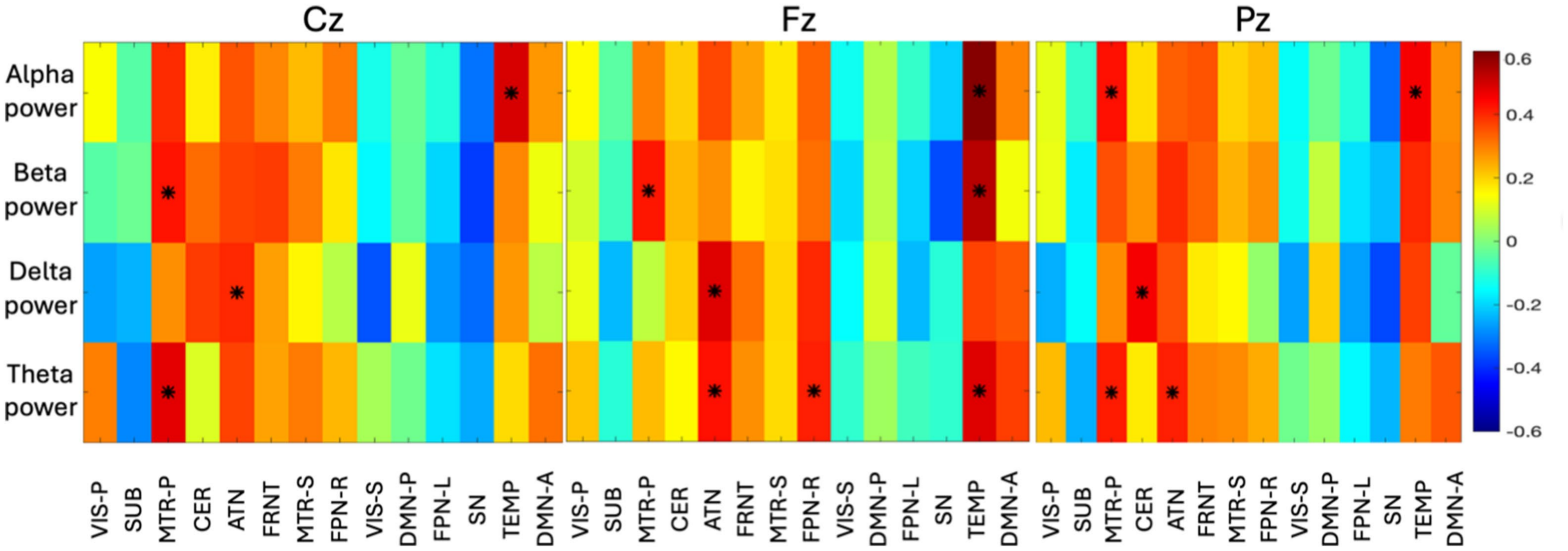
The group-level correlation map between time-varying spectral power in four bands and voxels of 14 networks are displayed here (correlation > abs|0.4| are identified with an asterisk). The principal component of all the subject specific correlation maps is shown as group-level correlation map. Three different correlation maps are presented for three different electrodes (Cz, Fz, Pz). The color bar indicates the correlation value ranging from −0.6 to +0.6.

In addition to the volume-based coupling, we measured the correlation between EEG spectral power and the first principal component of the voxel activity across time window. As evident from Figure 8, the temporal network is found to be correlated with Fz (fronto-central) with a wide range of frequencies (4–30 Hz). Theta, alpha, and beta (4 to 30 Hz) bands are prominently linked to the voxel activity of the temporal network, while the lower frequency (delta—0.5 to 4 Hz) was found to be less correlated (< abs|0.4|) across the time-window. The temporal network also shows high activity with the alpha band with Cz and Pz (central to parietal area). FPN-R is found to be highly correlated with the theta power (4 to 8 Hz) of Fz. In the ATN, the lower frequency (0.5 to 8 Hz) characteristics are observed at Fz, where delta (0.5 to 4 Hz) found to be active for Cz, and theta (4 to 8 Hz) found to be active for Pz. This can tell us that, the lower frequency of EEG is observed during spatial changes in the ATN network. CER is found to be correlated with low frequency (delta) of Pz, which summarizes that, CER is only connected to the parietal region. The high-frequency power (beta) was found to be highly correlated with MTR-P with Cz, which indirectly indicates that MTR-P is active during rest and connected to the frequency observed in motor imagination. The spatial changes in MTR-P are found to connected with theta and alpha spectral power at the parietal region (Pz).

## 4 Discussion

In this investigation, we developed a method to link fMRI (spatial resolution) with EEG (temporal resolution). As a result, the spatial dynamics of fMRI brain networks are found to be linked to power in EEG spectral bands which are also well-known features of mental state (Allen et al., 2018; Phadikar et al., 2024). The linking between fMRI spatial dynamic networks and EEG characteristics provides additional insights into the spatial–temporal dynamics of intrinsic connectivity networks. Rather than a static analysis, here we computed the spatial dynamics of the fMRI networks and explained how they vary in space, and this varying nature is synchronized with varying temporal activity of EEG. For this feasibility study, we used a model order of 20 to identify large-scale networks in the MOO-ICAR model. While the focus of the current study was on large scale networks, future work can use a high model order to extract more granular networks. Furthermore, future work can use multi-scale ICN template obtained in recent work to provide more comprehensive picture of changes in brain networks.

Alpha waves in EEG (8 to 13 Hz) are typically associated with a relaxed but awake state (Tuladhar et al., 2007). High alpha activity at the VIS-P and TEMP networks indicates strong evidence of calm, relaxed states. Previous studies showed that alpha (mu-rhythm) and beta activity in resting state may indicate the performance of motor imagery tasks (Lee et al., 2020; Wang et al., 2022; Zhang et al., 2015). Seeing significant alpha and beta activity at the MTR-P and CER network may be indirectly associated with states of relaxation with motor planning. The ATN network is crucial for maintaining focus, alertness, and selective attention. The association of delta and theta power with the ATN typically suggests reduced levels of attention and arousal. Increased delta activity at both Fz and Pz electrodes, correlated with the DMN-A network, suggests that the brain is in a state of reduced external engagement, focusing more on internal processes (Raman and Filho, 2024). Theta power at Cz and Pz shows strong association with both MTR-P and CER networks, it might indicate that theta oscillations play a role in coordinating motor and cognitive processes, however further studies are required to validate these findings. This could suggest a functional link between sensory processing, motor control, and executive functions. Theta power activity at Fz electrode, when correlated with the FPN-R and TEMP networks, suggests that frontal theta rhythms are involved in high-level cognitive processes like executive functioning, cognitive flexibility, and integration of information (Cao et al., 2022). We did not include gamma as resting-state EEG is often dominated by alpha (8–13 Hz) and theta (4–8 Hz) rhythms, especially in relaxed states, potentially overshadowing gamma activity (Allen et al., 2018).

This study focuses on investigating spatial changes in resting-state networks in relation to the EEG power spectrum. To reduce computational load, and minimize multiple comparisons, we prioritized midline electrodes, which approximately represent both hemispheres. Further, our results showed consistency across the electrodes likely since we considered small number of electrodes (Fz, Cz, Pz) at a specific location (midline). In future studies with larger sample sizes, we plan to focus on all the 32/64 electrodes and may observe the variation in the threshold.

While the spatial dynamic features of the above-mentioned networks are observed to be correlated with the spectral properties of EEG, the other networks that did not show such association, perhaps because they are not active during rest with eyes open. The analysis tells us about various frequency localization while resting state networks are evolving. We inferred an indirect link between mental states and fMRI networks based on observed correlations and previous studies. However, direct evidence requires further investigation. Future research should examine both the temporal and spatial dynamics of fMRI networks with eyes open and closed, as well as during behavioral tasks, to provide deeper insights. The identified links to the (eyes open) resting state reveal information about how brain networks interact and process information during rest. Future studies might leverage this approach to investigate whether disruptions in these interactions are associated with neurological and psychiatric disorders.

## Data Availability

The raw data supporting the conclusions of this article will be made available by the authors, without undue reservation.

## Footnotes

^1^ http://trendscenter.org/software/gift

^2^ http://sccn.ucsd.edu/eeglab

^3^ www.mathworks.com

^4^ https://fsl.fmrib.ox.ac.uk/eeglab/fmribplugin/

^5^ https://www.fil.ion.ucl.ac.uk/spm/software/spm12/
