# Correction: Integrating fMRI spatial network dynamics and EEG spectral power: insights into resting state connectivity

**DOI:** 10.3389/fnins.2026.1797621

**Published:** 2026-03-03

**Authors:** Souvik Phadikar, Krishna Pusuluri, Armin Iraji, Vince D. Calhoun

**Affiliations:** Tri-Institutional Center for Translational Research in Neuroimaging and Data Science (TReNDS), Georgia State University, Georgia Institute of Technology, and Emory University, Atlanta, GA, United States

**Keywords:** multimodal fusion, simultaneous EEG/fMRI, spatial dynamics, resting state networks, EEG spectra

There was a mistake in Table 1 as published. Citations mentioned in the columns 2 and 3 were swapped. The corrected Table 1 appears below.

**Table 1:** Simultaneous EEG/fMRI dataset description.

**Parameters**		**Dataset (Telesford et al., 2023)**	**Dataset (Gu et al., 2023)**	**Dataset (Wu et al., 2010)**	**Inhouse dataset**
Number of participants		22	33	25	10
Resting state duration (s)		600	600	420	420
fMRI	TR (ms)	2,100	2,100	2,000	2,000
	TE (ms)	24.6	25	39	30
	Matrix size	64 × 64	NA	64 × 64	64 × 64
	Voxel size (mm)	3.4 × 3.4 × 3.3	3 × 3 × 3	3.5 × 3.5 × 3.5	3.4 × 3.4 × 4
	Slices	38	35	27	32
EEG	Number of channels	64	32	32	32
	Impedances (kOhm)	< 20	< 20	< 5	< 5
	Sampling rate (KHz)	5	5	5	5

The original version of this article has been updated.

